# Assessment of multiple probiotic strains that protect *Montipora capitata* coral from infection by *Vibrio coralliilyticus*

**DOI:** 10.1128/spectrum.00443-26

**Published:** 2026-07-21

**Authors:** Blake Ushijima, Silvia Grant-Beurmann, Charles K. Gongaware, Patrick Videau, Claudia C. Häse

**Affiliations:** 1 Department of Biology and Marine Biology, University of North Carolina Wilmington 14621, Wilmington, North Carolina, USA; 2 Department of Obstetrics and Gynecology, School of Medicine, Duke University 3065, Durham, North Carolina, USA; 3 Department of Environmental Science, Policy, and Sustainability, Southern Oregon University 58757, Ashland, Oregon, USA; 4 Carlson College of Veterinary Medicine, Oregon State University 2694, Corvallis, Oregon, USA; Swansea University, Swansea, United Kingdom

**Keywords:** *Pseudoalteromonas*, coral, probiotic, *Vibrio*

## Abstract

Coral disease outbreaks threaten reef ecosystems, often leading to widespread mortality and declines in coral cover. Outbreaks of tissue loss diseases like acute *Montipora* white syndrome (aMWS) have impacted coral populations that include the Hawaiian rice coral (*Montipora capitata*). Multiple strains of *Vibrio coralliilyticus* are known pathogens, and strain OCN008 has been demonstrated as an etiological agent of aMWS in Hawaiʻi. Recent work has demonstrated that probiotic bacterial strains can be used to directly treat or prevent transmission (prophylaxis) of coral diseases. Based on their production of zones of inhibition and isolation from disease-resistant corals, *Pseudoalteromonas ardens* R96, *Pseudoalteromonas obscura* P94, strain Y97 (the genomic similarity to *Pseudoalteromonas piscicida* is presented), *Pseudoalteromonas umbrosa* B95, and *Vibrio tetraodonis* subsp. *pristinus* OCN044 were assessed for their ability to impair *V. coralliilyticus* OCN008 infection of *M. capitata* during laboratory infection trials. Individual inoculation of each of the five aforementioned strains on *M. capitata* fragments for 48 h prior to *V. coralliilyticus* OCN008 inoculation resulted in up to a 93.75% reduction in mortality. These results indicate that strains of *Pseudoalteromonas* and *Vibrio* can act as prophylactics to prevent *M. capitata* mortality from *V. coralliilyticus* OCN008 infection and provide tools to improve disease resilience for Pacific corals.

**IMPORTANCE:**

Coral disease outbreaks are a growing threat to the continued health of coral reefs, which are already vulnerable ecosystems. Strains of the bacterium *Vibrio coralliilyticus* are known to infect various coral species worldwide, predominantly causing tissue loss and death of the coral animal. Previous research has indicated that constituents from healthy coral microbiomes can act as probiotics to treat or prevent coral infections, and the discovery of effective probiotics is important in the effort to further develop mitigation tools for disease outbreaks. This work provides a demonstration of probiotic species that can protect coral from tissue loss infections by a strain of *Vibrio coralliilyticus* and is an example of probiotics developed for coral species in Hawaiʻi. This work provides new tools for probiotic-based coral protection and evidence for this research as a viable avenue to protect coral in their native environments.

## OBSERVATION

Tissue loss diseases (also referred to as white syndromes) are a devastating type of infection that can decimate reef-building coral populations by directly killing the coral animal (1–5). *Vibrio coralliilyticus* has been implicated in coral disease outbreaks (1, 2, 6, 7), and the pathogenic strain OCN008 is one of the etiological agents responsible for the tissue loss disease acute *Montipora* white syndrome (aMWS) in the Hawaiian rice coral *Montipora capitata* (7–9). While there are currently no effective *in situ* treatments for tissue loss disease outbreaks attributed to *V. coralliilyticus*, one promising treatment option is the application of beneficial microbes (i.e., probiotics) (10). Previous studies utilizing inhibition assays on laboratory media have demonstrated antibacterial activities from coral-associated *Pseudoalteromonas* and *Pseudovibrio* spp. against *V. coralliilyticus* (11, 12). Treatment of live coral specimens with the predatory *Halobacteriovorax* appeared to prevent the dysbiosis associated with *V. coralliilyticus* exposure (13), while another study indicated that a mixture of *Pseudoalteromonas*, *Cobetia*, and *Halomonas* spp. could prevent bleaching after exposure to other strains of this pathogen (14). Another recent work identified a *Ruegeria profundi* strain that provided protection against *V. coralliilyticus*-induced bleaching in *Acropora carduus*, but the study did not assess tissue loss (15). Similarly, a recent study demonstrated that the antimicrobial-producing *Pseudoalteromonas* sp. MCH1-7 strain could directly treat or protect Caribbean corals from stony coral tissue loss disease (SCTLD), which is not attributed to *V. coralliilyticus* (16). In this work, the probiotic potential of several *Pseudoalteromonas* and *Vibrio* strains to protect *M. capitata* against infection (prophylaxis) by *V. coralliilyticus* OCN008 was assessed.

**Fig 1:**
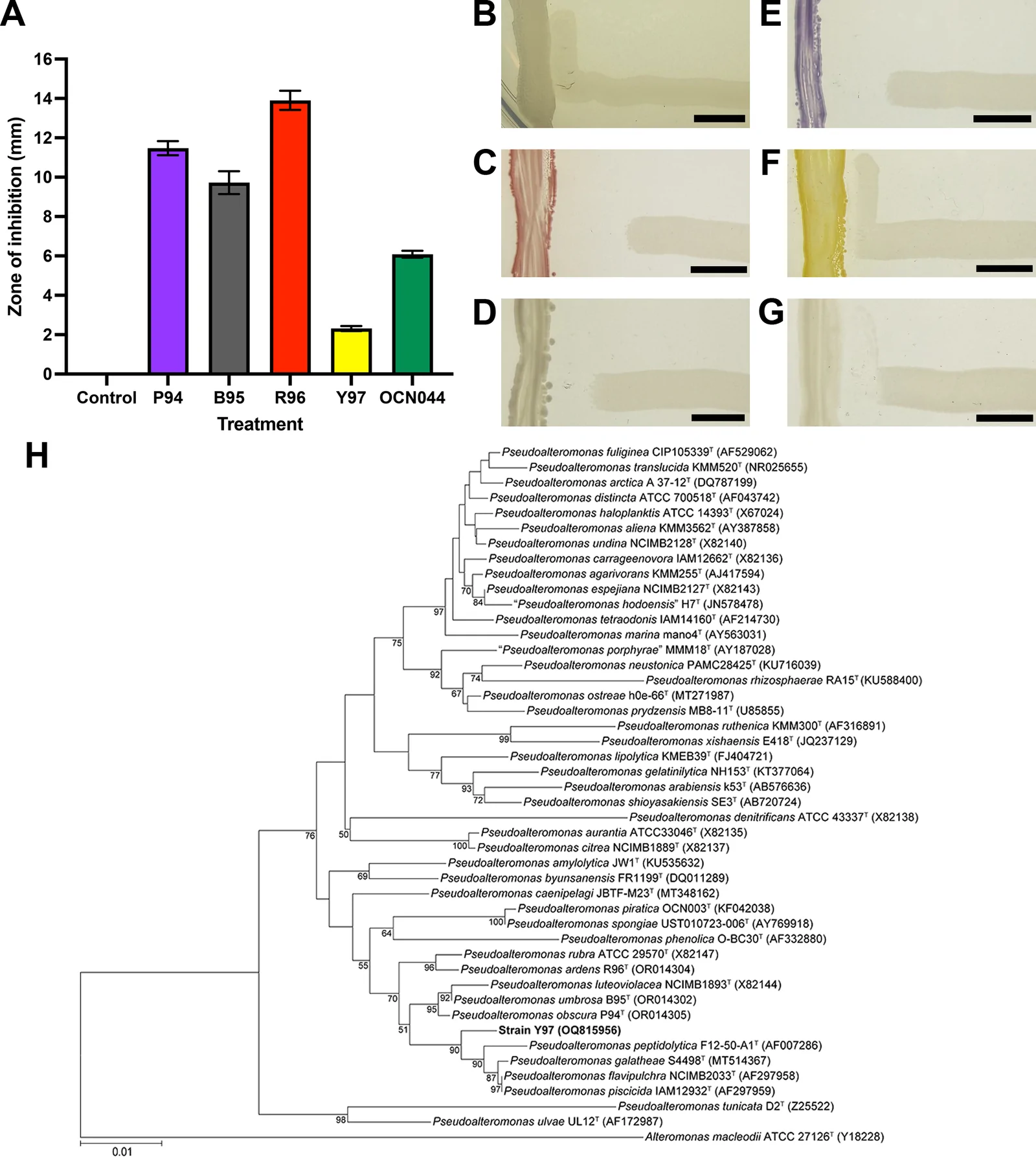
*Pseudoalteromonas ardens* R96, *Pseudoalteromonas obscura* P94, *Pseudoalteromonas piscicida* Y97, *Pseudoalteromonas umbrosa* B95, and *Vibrio tetraodonis* subsp. *pristinus* OCN044 inhibit the growth of *Vibrio coralliilyticus* OCN008 in plate-based assays as evidenced by the creation of zones of inhibition (ZOI). Growth inhibition was assessed for the ability of the *Alteromonas macleodii* OCN004 negative control (**B**), *P. ardens* R96 (**C**), *P. umbrosa* B95 (**D**), *P. obscura* P94 (**E**), *P. piscicida* Y97 (**F**), and *V. tetraodonis* subsp. *pristinus* OCN044 (**G**) to create a ZOI against *V. coralliilyticus* OCN008 utilizing T-streak assays. The ZOI is indicated by a lack of horizontal *V. coralliilyticus* OCN008 growth into the vertical strain. The black scale bar in each panel represents 1 cm. Digital measurement of the ZOI achieved against OCN008 is presented as the average of three inhibition assays ± standard deviation (**A**). Neighbor-joining dendrogram showing the phylogenic relationships between strains Y97 and related *Pseudoalteromonas* type strains based on 16S rRNA gene sequences (**H**). *Alteromonas macleodii* ATCC 27126^T^ was chosen as an outgroup. Bootstrap values >50% (1,000 replicates) are indicated at branch nodes. GenBank accession numbers for the 16S rRNA gene sequence of each reference strain are in parentheses. Bar, number of substitutions per nucleotide position.

*Pseudoalteromonas ardens* R96, *Pseudoalteromonas obscura* P94, and *Pseudoalteromonas umbrosa* B95 (hereafter referred to as R96, P94, and B95, respectively) were previously isolated from fragments of *Montipora capitata* that did not develop tissue loss after exposure to *V. coralliilyticus* OCN008 (8, 17). *Vibrio tetraodonis* subsp. *pristinus* OCN044 (hereafter referred to as OCN044) was isolated from healthy *Acropora cytherea* and used as a non-pathogenic control in previous disease studies (6), but was also found to inhibit the growth of neighboring bacterial colonies during isolation (18). Strain Y97 was a yellow colony isolated from a fragment of *A. cytherea* resistant to infection by *V. coralliilyticus* strain OCN014 (6, 17). A 16S rRNA gene sequenced-based phylogeny indicated that Y97 was affiliated with the genus *Pseudoalteromonas* in a branch containing *Pseudoalteromonas peptidolytica*, *Pseudoalteromonas galatheae*, *Pseudoalteromonas flavipulchra*, and *Pseudoalteromonas piscicida* in (Fig. 1H; refer to the Supplemental Material for details on the experimental methodology). As this grouping in the phylogenetic tree was not well resolved, the Average Nucleotide Identity (ANI) and *in silico* DNA–DNA hybridization (isDDH) between strain Y97 and all potentially related type strains in the cluster were determined (Table S1). The ANI and isDDH values between strain Y97 and *P. piscicida* JCM 20779^T^ were >97 and >70%, respectively, which indicated that strain Y97 is affiliated with *P. piscicida* (hereafter referred to as Y97). Based on the nature of the coral fragments from which the above strains were isolated, each strain was assessed for its ability to inhibit the growth of *V. coralliilyticus* OCN008. Utilizing a plate-based T-streak method to assess growth inhibition, each isolate created a zone of inhibition (ZOI) for *V. coralliilyticus* OCN008, whereas no ZOI was apparent from the negative control (Fig. 1B through G). Measurement of the ZOI found that R96 produced the greatest ZOI, while Y97 yielded the smallest (Fig. 1A). Previous work indicated that multiple members of the *Pseudoalteromonas* genus produce antimicrobial compounds, and pigmented strains may represent resources for secondary metabolite production (19, 20). The isolation of five strains with inhibitory activities against *V. coralliilyticus* OCN008 from healthy coral fragments and those resistant to infection by *V. coralliilyticus* necessitated testing them as potential coral probiotics.

**Fig 2:**
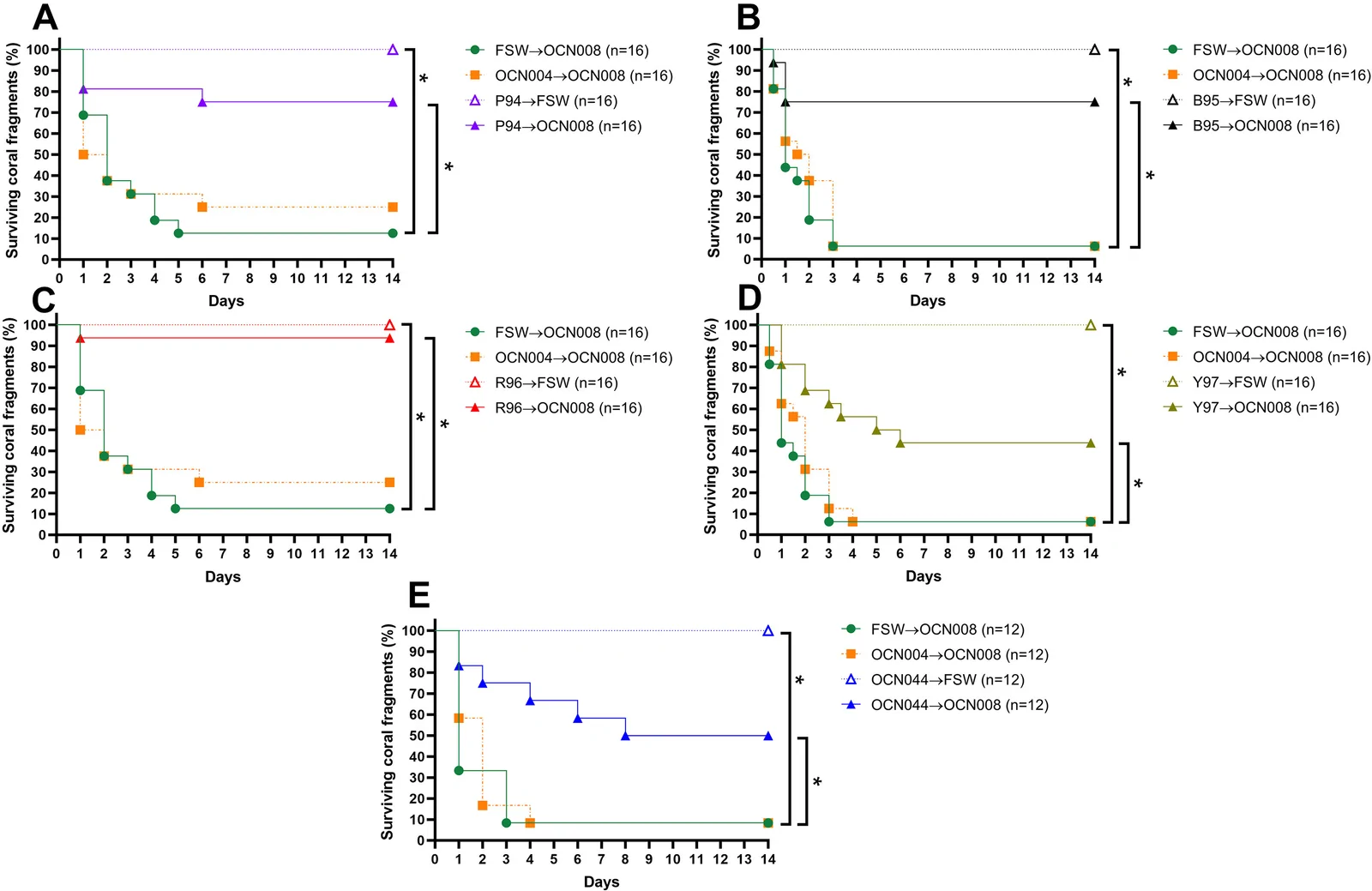
Kaplan–Meier survival curves of *Montipora capitata* fragments treated with the probiotic strains *Pseudoalteromonas obscura* P94 (**A**), *Pseudoalteromonas umbrosa* B95 (**B**), *Pseudoalteromonas ardens* R96 (**C**), *Pseudoalteromonas piscicida* Y97 (**D**), or *Vibrio tetraodonis* subsp. *pristinus* OCN044 (**E**) to protect against infection by *Vibrio coralliilyticus* OCN008. The order of treatments with FSW, *Alteromonas macleodii* OCN004 (negative control bacterium; OCN004), and *V. coralliilyticus* OCN008 (pathogen; OCN008) are included in the legends to the right of each graph demonstrating the survival outcomes. Brackets with an asterisk indicate conditions that are significantly different (*P* < 0.01) based on log-rank (Mantel–Cox) tests, and corresponding values are presented in Table S2.

Previous work has demonstrated that laboratory infection trials of *Montipora capitata* with bacterial pathogens can be used to evaluate etiological agents of disease and mechanisms underlying virulence (6, 8, 21). During laboratory infection trials, *M. capitata* fragments maintained in laboratory aquaria were exposed to bacterial pathogens, and disease progression was monitored over time, which generally results in tissue loss and coral fragment death from *V. coralliilyticus* OCN008 infection. To determine the capacity of the strains listed above to act as coral probiotics (specifically as prophylactics), *M. capitata* fragments were inoculated with the potential probiotic strains for 48 h during pre-treatment, transferred to new aquaria, challenged with the known pathogen *Vibrio coralliilyticus* OCN008 or a negative control in a post-treatment, and then monitored for tissue loss lesion development and mortality. When 10^8^ CFU/mL of *V. coralliilyticus* OCN008 was inoculated onto *M. capitata* fragments without probiotic pre-treatment, 87.50–93.75% mortality was observed within 5 days post-inoculation (Fig. 2; Table S2), which is similar to previous work (6, 8, 21). The results of statistical comparisons are presented in Table S2. Previous studies utilized *Alteromonas macleodii* OCN004 as a negative control bacterium that did not infect *M. capitata* fragments at the same dose as bacterial pathogens (8, 22–24). Pre-treatment of *M. capitata* fragments with *A. macleodii* OCN004 as a bacterial negative control for probiotic inoculation for 48 h prior to *V. coralliilyticus* OCN008 post-treatment resulted in 75.00–93.75 % mortality, which was not significantly different than mortality levels following *V. coralliilyticus* OCN008 inoculation alone in each trial (Table S2). In contrast, pre-treatment of *M. capitata* fragments with P94, B95, R96, Y97, or OCN044 for 48 h prior to pathogen introduction reduced mortality from *V. coralliilyticus* OCN008 by 37.50–93.75%, which was significantly lower than inoculation with *V. coralliilyticus* OCN008 alone or following pre-treatment with the *A. macleodii* OCN004 bacterial control. The greatest level of protection resulted from pre-treatment with R96 (93.75% reduced mortality), followed by B95 (68.75% reduced mortality), P94 (62.50% reduced mortality), and OCN044 (41.67% reduced mortality), and the lowest level of protection was conferred by Y97 (37.50% reduced mortality). Neither the addition of filtered seawater (FSW) as a negative control to account for changes in aquaria water level nor the addition of the probiotic strains alone resulted in coral mortality (Table S2). A comparison of plate-based antimicrobial ZOI production and decreases observed in OCN008 infection following probiotic pre-treatment compared to negative controls found a significant positive correlation (Pearson’s *r* = 0.94; *P* = 0.02), which indicates that strain-specific levels of antimicrobial production may be a driver of probiotic activity *in situ*. These results indicate that inoculation of P94, B95, R96, Y97, or OCN044 onto *M. capitata* fragments prior to pathogen challenge decreases coral mortality compared to conditions where these strains were not included, which indicates that these strains are viable probiotics and can act as prophylactics.

To date, multiple outbreaks of *Montipora* white syndrome have been documented in Hawaiʻi, and there are little to no treatment options to prevent infection in corals. These results present *P. ardens* R96, *P. obscura* P94, *P. piscicida* Y97, *P. umbrosa* B95, and *V. tetraodonis* subsp. *pristinus* OCN044 as probiotics that can lower mortality from aMWS and represent probiotic strains to show efficacy against coral tissue loss caused by *V. coralliilyticus*. The findings presented here provide a potential path forward to define and test further treatment options to mitigate the damage caused by *V. coralliilyticus*.

## Data Availability

The 16S rRNA gene sequence for *Pseudoalteromonas piscicida* Y97 was deposited in DDBJ/ENA/GenBank under accession number OQ815956. The draft genome sequence for *P. piscicida* Y97 is deposited in DDBJ/ENA/GenBank under accession number JASGWW000000000, while raw reads are deposited in the SRA under accession number SRR25510053. *P. ardens* R96 (DSM 114998, LMG 32870), *P. obscura* P94 (DSM 114996, LMG 32871), *P. umbrosa* B95 (DSM 114997, LMG 32872), and *V. tetraodonis* subsp. *pristinus* OCN044 (LMG 31895, DSM 111778) are available from type culture collections, while *P. piscicida* Y97 may be made available upon request.

## SUPPLEMENTAL MATERIAL

Supplemental Material spectrum.00443-26-s0001.pdf Supplemental methods; Tables S1 and S2.

ASM does not own the copyrights to Supplemental Material that may be linked to, or accessed through, an article. The authors have granted ASM a non-exclusive, world-wide license to publish the Supplemental Material files. Please contact the corresponding author directly for reuse.
