# Supplementary material for: Assessment of multiple probiotic strains that protect Montipora capitata coral from infection by Vibrio coralliilyticus
Source: Microbiol Spectr. 2026 Jul 21;14(9):e00443-26. doi: 10.1128/spectrum.00443-26 (PMC13532223; doi:10.1128/spectrum.00443-26)
Supplement: Supplementary file 1 [file spectrum.00443-26-s0001.pdf]

Assessment of multiple probiotic strains that protect *Montipora capitata* coral from infection by *Vibrio coralliilyticus*

Blake Ushijima[a,#], Silvia Grant-Beurmann[b], Charles K. Gongaware[a], Patrick Videau[c], Claudia C. Häse[d,#]

[a]Department of Biology and Marine Biology, University of North Carolina Wilmington, Wilmington, North Carolina, United States of America

[b]Department of Obstetrics and Gynecology, School of Medicine, Duke University, Durham, North Carolina, United States of America

[c]Department of Environmental Science, Policy, and Sustainability, Southern Oregon University, Ashland, Oregon, United States of America

[d]Carlson College of Veterinary Medicine, Oregon State University, Corvallis, Oregon, United States of America

[#]Corresponding authors: Blake Ushijima and Claudia C. Häse

E-mails: ushijimab@uncw.edu (BU), Claudia.Hase@oregonstate.edu (CCH)

Supplemental Materials and Methods

Strain isolation and culture conditions

Strain Y97 was isolated from a fragment of *A. cytherea* resistant to *V. coralliilyticus* OCN014 infection from Palmyra Atoll, Northern Line Islands. Briefly, using a sterile pipette, surface mucus was collected from coral fragments suspended outside of the water and then plated on a sea water agar (SWA) prepared as previously described (1). The plated mucus was incubated at 28 °C overnight and colonies were purified on the same medium. For all subsequent experiments conducted in this work, strains were cultured at 21 °C or 28 °C on GASW buffered with 50 mM Tris with the pH adjusted to 8.3 with concentrated HCl before autoclaving (hereafter GASW-Tris) (2), solidified with 1.5 % agar (w/v), or shaken at 125 RPM for broth cultures.

Genome sequencing and taxonomic analysis

Genomic DNA was isolated from strain Y97 via phenol-chloroform extraction and used in a PCR to amplify a fragment of the 16S rRNA gene with the primers 8F (5'-AGAGTTTGATCCTGGCTCAG-3') and 1492R (5'- TACGGYTACCTTGTTACGACTT -3') (3, 4). The amplification product was purified with the Wizard SV Gel and PCR Clean-up System (Promega, Madison, WI) and sequenced by Sanger Sequencing in the Biotechnology Center at Southern Oregon University using the same primers. The 16S rRNA gene sequence was assessed for similarity using BLAST within the NCBI website with uncultured/environmental samples excluded (5). The 16S rRNA gene sequence of strain Y97 and those from related strains were aligned using the MUSCLE algorithm (6), trimmed to 1350 bp, and a neighbor-joining phylogenetic tree was prepared using the MEGA12.1 software package (7).

For genome sequencing, a GASW-Tris agar plate of strain Y97 derived from a single colony was sent to SeqCenter, LLC (Pittsburgh, PA, USA) for genomic DNA isolation with the Qiagen DNeasy Blood and Tissue kit according to the manufacturer's instructions (Hilden, Germany). DNA libraries were prepared with the Illumina DNA Prep kit and IDT 10 bp UDI indices and sequenced on an Illumina NextSeq 2000 (8). The 151-bp paired-end raw Illumina reads were trimmed with bbduk (version 38.73; https://sourceforge.net/projects/bbmap/) and assembled into a draft genome with SPAdes v. 3.15.0 using the "--careful" option and specifying kmers of 21, 33, 55, 77, 99, and 121 (9). Average Nucleotide Identity (ANI) values were determined with FastANI in Proksee using the default parameters (10, 11). DNA-DNA Hybridization (isDDH) was conducted *in silico* using GGDC 3.0 with the default parameters (12, 13). The species boundary cutoffs for genomic indices used in this study were 95-96 % ANI (10, 14-17) and 70 % isDDH (12, 18).

Growth inhibition assays

A single line of the putative probiotic strains *Pseudoalteromonas ardens* R96, *Pseudoalteromonas obscura* P94, *Pseudoalteromonas umbrosa* B95, *Pseudoalteromonas piscicida* Y97, and *Vibrio tetraodonis* subsp. *pristinius* OCN044, as well as *Alteromonas macleodii* OCN004 as a negative control, was streaked down the center of GASW-Tris plates and incubated at 23 °C for three days. An overnight culture of *Vibrio coralliilyticus* OCN008 was washed three times with artificial seawater (ASW) and diluted to a final OD_{600} = 0.8. Utilizing sterile cotton-tipped applicators, *V. coralliilyticus* OCN008 was streaked directly up to the line of growth formed by the probiotic strains, and the plates were incubated at 23 °C for 24 h. The formation of a zone of inhibition was assessed visually by comparison to the negative control

and quantified using the digital caliper function in ImageJ v.1.53 with the scale defined via a ruler included alongside plates in the raw images (19).

Coral collection, husbandry, and infection experiments

Fragments of the coral *A. cytherea* were collected by authorized individuals from a reef at Palmyra Atoll, Northern Line Islands under special activity permits 12533-11025, 12533-12025, 12533-14025 granted by the U.S. Fish and Wildlife Service. Descriptions of the *A. cytherea* fragment collection and isolation of their associated microbes were previously described (1). Fragments of the coral *M. capitata* were collected under special activities permits 2015-48 and 2018-03 issued by the Hawai‘i Department of Land and Natural Resources, Division of Aquatic Resources, by authorized individuals. The fragments were approximately 3 x 3 cm in size from colonies on the fringing reef surrounding the island Moku o Lo‘e in Kāne‘ohe Bay, O‘ahu, Hawai‘i, USA and were from the orange, plating *M. capitata* morphology for consistency. Fragments were kept under ambient light and temperature in a flow-through water table for two days to allow for healing before use.

Coral experiments used a previously designed setup and were kept at 27 ºC under ambient sunlight (20), but with some modifications as described below. To ensure that multiple genets were assessed in this work, fragments were collected from 12-16 different colonies, the number of which is denoted by the *n*-value in Table S2. The coral experiments followed a block design, where each experimental block used four fragments from the same colony while no separate blocks used fragments from the same colony. Each experimental block consisted of four combinations of “pre-treatments” and “post-treatments” that were done in separate tanks (Table S2). The four combinations of treatments were: 1) filtered seawater (FSW) only pre-treatment

then a *V. coralliilyticus* OCN008 post-treatment (OCN008 control), 2) *Alteromonas macleodii* OCN004 (negative bacterial control) pre-treatment then a *V. coralliilyticus* OCN008 post-treatment (OCN004 control), 3) probiotic candidate pre-treatment then a FSW post-treatment, and 4) probiotic candidate pre-treatment then a *V. coralliilyticus* OCN008 post-treatment. The FSW used for all experiments was acquired from the Kewalo Marine Laboratory system, which passed seawater through a 0.5 µm-pore bag filter, then a 0.22 µm-pore membrane filter. This FSW was transported in 20 L polypropylene carboys and was further sterilized within the carboys via autoclaving for 60 min at 121 ºC. Each experimental block used FSW from the same collection timepoint to reduce variability with salinity, which was checked to ensure 34-35 ppt before use and adjusted with autoclaved, ultrapure water as needed. For each pre-treatment, no more than four coral fragments were housed in a glass aquarium filled with 7 L of seawater with their respective treatment applied. Pre-treatments (FSW, *A. macleodii* OCN004, or a probiotic candidate) were a single inoculation and the corals were left in these pre-treatment tanks for 48 h without water changes. All bacterial strains were prepared as previously described and inoculated to a final concentration of 10^8 CFU/ml of tank water (20). This was achieved by concentrating the strains to 10^{10} CFU/ml and adding 10 mL/L of tank water. After 48 h, the corals were removed from their pre-treatment tanks with sterile forceps and individually placed into plastic tanks filled with 4 L of FSW with care taken to minimize liquid carryover. These smaller tanks were where the corals received their post-treatment (FSW or *V. coralliilyticus* OCN008) and were kept for the remainder of the experiment. All *V. coralliilyticus* OCN008 treatments were prepared as previously described and inoculated to a final concentration of 10^8 CFU/ml of tank water (20). Partial water changes were done every 2 days starting on day 5 post-treatment. Due to the limitations on coral collection, the *V. coralliilyticus* OCN008 and *A. macleodii* OCN004

controls were shared between the *Pseudoalteromonas obscura* P94 and *Pseudoalteromonas ardens* R96 trials and between the *Pseudoalteromonas umbrosa* B95 and *Pseudoalteromonas piscicida* Y97 trials. The determination of "mortality" in survival curves was considered to be any coral fragment with greater than 30% visible tissue loss on the surface (2, 21); previous studies indicate that fragments with this level of tissue damage do not survive until the next day in this state (1, 2, 20-22). Statistical analyses were conducted in GraphPad Prism (San Diego, CA, USA).

Table S1. Genomic comparisons between strain Y97 (GCA_030710255.1) and strains that cluster with it in Figure 1. Average Nucleotide Identity (ANI) and *in silico* DNA-DNA Hybridization (isDDH) comparisons are presented.

**Strain**	**FastANI (%)**	**isDDH (%)**
Pseudoalteromonas flavipulchra ATCC BAA-314 ^{T} (GCA_000814665.1)	96.37	69.9
Pseudoalteromonas galatheae S4498^{T} (GCA_005886105.2)	92.54	48.1
Pseudoalteromonas peptidolytica DSM 14001^{T} (GCA_012641745.1)	83.52	24.1
Pseudoalteromonas piscicida JCM 20779^{T} (GCA_000238315.4)	97.39	77.9

Table S2. Coral infection experiment treatment outline and results, which are visualized in Figure 2. The *n*-values refer to the number of colonies that were used for each experiment.

Experiment	Pre-treatment	Post-treatment	Percent Mortality (%)	Mean survival (days)[*]	*n*-value	*p*-value[**]
1	FSW	OCN008	91.67	2.75	12	0.8370 (vs. OCN004 control)
	OCN004	OCN008	91.67	2.58	12	0.8370 (vs. OCN008 control)
	OCN044	FSW	0.00	14.00	12	<0.0001 (vs. OCN008 control)
	OCN044	OCN008	50.00	8.83	12	0.0053 (vs. OCN008 control)
2	FSW‡	OCN008‡	87.5	3.68	16	0.7097 (vs. OCN004 control)
	OCN004‡	OCN008‡	75.00	4.81	16	0.7097 (vs. OCN008 control)
	P94^{T}	FSW	0.00	14.00	16	<0.0001 (vs. OCN008 control)
	P94^{T}	OCN008	25.00	11.06	16	0.0004 (vs. OCN008 control)
3	FSW†	OCN008†	93.75	2.18	16	0.4397 (vs. OCN004 control)
	OCN004†	OCN008†	93.75	2.50	16	0.4397 (vs. OCN008 control)
	B95^{T}	FSW	0.00	14.00	16	<0.0001 (vs. OCN008 control)
	B95^{T}	OCN008	25.00	10.72	16	0.0002 (vs. OCN008 control)
4	FSW‡	OCN008‡	87.5	3.68	16	0.7097 (vs. OCN004 control)
	OCN004‡	OCN008‡	75.00	4.81	16	0.7097 (vs. OCN008 control)
	R96^{T}	FSW	0.00	14.00	16	<0.0001 (vs. OCN008 control)
	R96^{T}	OCN008	6.25	13.19	16	<0.0001 (vs. OCN008 control)
5	FSW†	OCN008†	93.75	2.18	16	0.4397 (vs. OCN004 control)
	OCN004†	OCN008†	93.75	2.50	16	0.4397 (vs. OCN008 control)
	Y97	FSW	0.00	14.00	16	<0.0001 (vs. OCN008 control)
	Y97	OCN008	56.25	7.66	16	0.0011 (vs. OCN008 control)

Abbreviations: FSW, filtered seawater; OCN004, negative control bacterium *Alteromonas* sp. OCN004; OCN008, coral pathogen *Vibrio coralliilyticus* OCN008; OCN044, *Vibrio tetraodonis* subsp. *pristinus* OCN044; P94^{T}, strain P94^{T}; B95^{T}, strain B95^{T}; R96^{T}, strain R96^{T}; Y97, strain Y97.

[*]Maximum length of experiments was 14 days.

**Based on log-rank (Mantel-cox) test compared to indicated control in parathesis.
†Control treatments were shared between experiments.
‡Control treatments were shared between experiments.

References

1. Ushijima B, Videau P, Poscablo D, Stengel JW, Beurmann S, Burger AH, Aeby GS, Callahan SM. 2016. Mutation of the *toxR* or *mshA* genes from *Vibrio coralliilyticus* strain OCN014 reduces infection of the coral *Acropora cytherea*. Environ Microbiol 18:4055-4067.
2. Ushijima B, Häse CC. 2018. Influence of chemotaxis and swimming patterns on the virulence of the coral pathogen *Vibrio coralliilyticus*. J Bacteriol 200:e00791-17.
3. Aebischer T, Fischer A, Walduck A, Schlötelburg C, Lindig M, Schreiber S, Meyer TF, Bereswill S, Göbel UB. 2006. Vaccination prevents *Helicobacter pylori*-induced alterations of the gastric flora in mice. FEMS Immunol Med Microbiol 46:221-9.
4. Sambrook J. 2001. Molecular cloning : a laboratory manual / Joseph Sambrook, David W. Russell. Cold Spring Harbor Laboratory, Cold Spring Harbor, N.Y.
5. Altschul SF, Gish W, Miller W, Myers EW, Lipman DJ. 1990. Basic local alignment search tool. J Mol Biol 215:403-10.
6. Edgar RC. 2004. MUSCLE: multiple sequence alignment with high accuracy and high throughput. Nucleic Acids Res 32:1792-1797.
7. Stecher G, Suleski M, Tao Q, Tamura K, Kumar S. 2025. MEGA 12.1: Cross-platform release for macOS and Linux operating systems. J Mol Evol 94:14-18.
8. Baym M, Kryazhimskiy S, Lieberman TD, Chung H, Desai MM, Kishony R. 2015. Inexpensive multiplexed library preparation for megabase-sized genomes. PLoS One 10:e0128036.
9. Prjibelski A, Antipov D, Meleshko D, Lapidus A, Korobeynikov A. 2020. Using SPAdes de novo assembler. Curr Protoc Bioinformatics 70:e102.
10. Jain C, Rodriguez-R LM, Phillippy AM, Konstantinidis KT, Aluru S. 2018. High throughput ANI analysis of 90K prokaryotic genomes reveals clear species boundaries. Nat Commun 9:5114.
11. Grant JR, Enns E, Marinier E, Mandal A, Herman EK, Chen C-y, Graham M, Van Domselaar G, Stothard P. 2023. Proksee: in-depth characterization and visualization of bacterial genomes. Nucleic Acids Research 51:W484-W492.
12. Meier-Kolthoff JP, Auch AF, Klenk HP, Göker M. 2013. Genome sequence-based species delimitation with confidence intervals and improved distance functions. BMC Bioinformatics 14:60.
13. Meier-Kolthoff JP, Carbasse JS, Peinado-Olarte RL, Göker M. 2021. TYGS and LPSN: a database tandem for fast and reliable genome-based classification and nomenclature of prokaryotes. Nucleic Acids Res 50:D801-D807.
14. Varghese NJ, Mukherjee S, Ivanova N, Konstantinidis KT, Mavrommatis K, Kyrpides NC, Pati A. 2015. Microbial species delineation using whole genome sequences. Nucleic Acids Res 43:6761-71.
15. Chun J, Oren A, Ventosa A, Christensen H, Arahal DR, da Costa MS, Rooney AP, Yi H, Xu XW, De Meyer S, Trujillo ME. 2018. Proposed minimal standards for the use of genome data for the taxonomy of prokaryotes. Int J Syst Evol Microbiol 68:461-466.
16. Ciufo S, Kannan S, Sharma S, Badretdin A, Clark K, Turner S, Brover S, Schoch CL, Kimchi A, DiCuccio M. 2018. Using average nucleotide identity to improve taxonomic assignments in prokaryotic genomes at the NCBI. Int J Syst Evol Microbiol 68:2386-2392.

17. Riesco R, Trujillo ME. 2024. Update on the proposed minimal standards for the use of genome data for the taxonomy of prokaryotes. Int J Syst Evol Microbiol 74:0063000.
18. Goris J, Konstantinidis KT, Klappenbach JA, Coenye T, Vandamme P, Tiedje JM. 2007. DNA-DNA hybridization values and their relationship to whole-genome sequence similarities. Int J Syst Evol Microbiol 57:81-91.
19. Schneider CA, Rasband WS, Eliceiri KW. 2012. NIH Image to ImageJ: 25 years of image analysis. Nat Methods 9:671-675.
20. Ushijima B, Videau P, Burger AH, Shore-Maggio A, Runyon CM, Sudek M, Aeby GS, Callahan SM. 2014. *Vibrio coralliilyticus* strain OCN008 is an etiological agent of acute *Montipora* white syndrome. Appl Environ Microbiol 80:2102-9.
21. Ushijima B, Saw JH, Videau P, Häse CC. 2022. Comparison of *Vibrio coralliilyticus* virulence in Pacific oyster larvae and corals. Microbiology 168:001169.
22. Ushijima B, Richards GP, Watson MA, Schubiger CB, Häse CC. 2018. Factors affecting infection of corals and larval oysters by *Vibrio coralliilyticus*. PLoS One 13:e0199475.
